# Narrative review on microbiota and sepsis: the host’s betrayal?

**DOI:** 10.1007/s11739-025-04215-8

**Published:** 2025-12-02

**Authors:** Matteo Guarino, Agostino Di Ciaula, Piero Portincasa, Roberto De Giorgio

**Affiliations:** 1 Department of Translational Medicine, St. Anna University Hospital of Ferrara, University of Ferrara, Via A. Moro 8, 44124 Ferrara, Italy; 2 Emergency Department, St. Anna University Hospital of Ferrara, Ferrara, Italy; 3 Department of Precision and Regenerative Medicine and Ionian Area (DiMePre-J), Clinica Medica “A. Murri”, University of Bari “Aldo Moro”, Bari, Italy

**Keywords:** Dysbiosis, Faecal microbiota transplantation, Gut barrier, Immune system, Sepsis

## Abstract

Sepsis remains a leading cause of morbidity and mortality worldwide. Increasing evidence suggests that the gut microbiota, long considered a “less relevant” to human body health, it plays a crucial role in the pathophysiology of sepsis. Disruption of the host–microbe balance contributes to impaired barrier integrity, microbial translocation, and dysregulated immune responses. This perspective raises the possibility that dysbiosis is not merely a consequence of critical illness, rather an active driver of septic progression. This narrative review explores the relationship between sepsis and gut microbiome. PubMed, Scopus, and EMBASE were searched from inception to September 2025. Recent studies have highlighted the triangular interplay between the intestinal barrier, gut microbiota, and immune system. Altered microbial composition and increased permeability foster systemic inflammation and immune dysfunction. Biomarkers such as diamine oxidase and intestinal fatty acid-binding protein are emerging as promising indicators of gut injury. Experimental therapies (i.e., faecal microbiota transplantation, targeted probiotics, prebiotics, postbiotics, and personalized antibiotic regimens guided by microbial profiling) provide potential to modulate host–microbe interactions. Integration of microbiome analysis with multi-omics and advanced bioinformatics may enable stratification of septic patients by microbial signatures, paving the way for precision medicine approaches. Modulation of gut microbiota represents a novel therapeutic frontier in sepsis. Conceptualizing sepsis as a disease of disrupted host–microbe symbiosis may unravel new diagnostic and therapeutic strategies. Future research should aim at prioritizing high-quality trials, innovative designs, and equitable implementation to target microbiota to improve survival and recovery in patients with sepsis.

## Introduction

Sepsis and shock are one of the most daunting challenges in modern medicine [1]. Despite decades of research, septic shock mortality ranges between 30 and 50%, and therapeutic innovations remains largely unsatisfactory [1, 2]. For years, the pathophysiology of sepsis has been framed through the lens of systemic inflammation, endothelial dysfunction, and immune exhaustion [3, 4]. However, recent evidence points toward a critical role exerted by the gut microbiota and related changes, i.e., dysbiosis, which can affect the clinical profile and outcome of sepsis/septic shock [5–8]. Once considered of marginal importance, the human microbiome is now recognized as a dynamic and metabolically active organ with the potential to shape systemic physiology as profoundly as the heart, liver, or kidneys [9, 10]. In sepsis, gut microbiota may not simply be a collateral damage, rather an active contributor of the host’s downfall [6, 7].

The gastrointestinal tract is key to the development of critical illness [6]. Indeed, loss of epithelial integrity, increased permeability, and bacterial translocation have long been invoked as factors triggering and perpetuating systemic inflammation which, in turn, is the common denominator underlying a variety of clinical phenotypes, including sepsis/septic shock [11]. The inflammatory activation would not be understandable without considering the microbial communities residing into the lumen or laying on the mucus layer of the intestinal surface [5–15]. Dysbiosis, a term used to indicate the disruption of microbial richness and diversity, is now documented as an underlying feature among critically ill patients [6, 15]. A few days of intensive care unit (ICU) admission can affect microbial richness, reduce commensals, and favour the increase of opportunistic pathogens, such as *Clostridiales*, *Enterococcus* or *Pseudomonas* [16, 17]. This shifts the gut from a site of symbiosis to a reservoir of harmful germs, fuelling systemic inflammation and secondary infections [17]. The correlation between dysbiosis and sepsis onset and severity is more than circumstantial [7, 18–20]. Preclinical models demonstrate that antibiotic-induced depletion of the microbiota renders experimental animals more susceptible to overwhelming infection [18, 20]. Conversely, restoration of microbial balance, through probiotics, prebiotics or faecal microbiota transplantation, can mitigate inflammatory responses and improve survival in experimental sepsis [19]. Human data, though limited, echo these findings: reduced alpha diversity and enrichment of pathobionts correlate with poor outcomes, prolonged ICU stay, and higher risk of multidrug-resistant colonization [21]. Such evidence raises an unsettling question: is the progression to septic shock driven not only by the invading pathogen but also by the host’s own microbiota?

The relationship between environmental factors, microbiota and immunity provides a compelling mechanistic framework. Microbial metabolites, such as short-chain fatty acids (SCFAs), regulate T-cell differentiation, modulate cytokine release, and sustain epithelial integrity [14, 19, 20, 22]. Dysbiosis deprives the host of these regulatory signals, tipping the immune system toward a pro-inflammatory and dysfunctional state [21]. Meanwhile, pathogen-associated molecular patterns (PAMPs) from overgrowing species continuously stimulate innate immune receptors, sustaining the vicious cycle of inflammation [15]. In this context, the gut is not a bystander but a central stage, where microbial and immune factors dictate the trajectory of sepsis. Emerging clinical biomarkers support this conceptual shift. Circulating markers of intestinal injury, such as intestinal fatty acid binding protein (I-FABP) or diamine oxidase (DAO), rise in parallel with dysbiosis and predict poor outcomes. Microbiome profiling studies suggest that certain microbial signatures may serve as prognostic indicators, potentially identifying patients at risk of progression from sepsis to shock [23]. If validated, these findings could redefine early risk stratification, moving beyond classic systemic biomarkers, such as procalcitonin or presepsin, to include microbial fingerprints.

Acknowledging the role of the gut microbiota in sepsis also forces us to reconsider either prevention or therapeutic strategies. Standard care often relies on broad-spectrum antibiotics, which are lifesaving but also detrimental for microbial diversity [1]. Indeed, drugs that can suppress pathogens may also dismantle the protective functions of commensals, opening the door to colonization by resistant organisms and amplifying systemic inflammation. This paradox highlights the urgent need for precision: antibiotics guided not only by pathogen susceptibility but also by the preservation of microbial balance. Similarly, innovative approaches such as faecal microbiota transplantation (FMT), targeted probiotics, or engineered postbiotics raise the possibility of actively manipulating the gut microbiome to restore host resilience [5].

Sepsis has always been viewed as the loss of the equilibrium between host defences and invading microbes [6]. Yet, this dichotomy may be too simplistic. A growing body of evidence suggests that the host’s own microbiota can be affected by the stress of critical illness. The intestinal barrier, the immune system, and the microbiome form a triad whose collapse may result into septic shock [12, 13]. If this perspective holds true, it carries profound implications. Beyond supportive care and anti-infective therapy, the management of sepsis may 1 day include strategies aimed at preserving or restoring microbial homeostasis. Analysis of individual risk factors, microbiome-guided risk stratification, tailored antibiotics, and targeted microbial therapies could emerge as integral parts of precision medicine in the ICU [5, 17]. The challenge now is to move from association to causation, from description to prevention and intervention, and from neglect to integration. This narrative review tackling the critical topic of gut microbiota in sepsis is thought as a conceptual basis to further study aimed at better understanding this condition and improving its management.

### Search strategy

We performed a comprehensive literature search using PubMed, EMBASE, and Scopus from database inception to September 2025. The search strategy combined MeSH terms and free-text keywords as follows: "Sepsis" OR "Septicemia" OR "Septic Shock" AND "Gastrointestinal Microbiome" OR "Intestinal Microbiota" OR "Gut Microbiota" OR "Gut Microbiome" OR "Intestinal Microbiome" OR "Enteric Microbiota". Additional filters were applied to include only studies involving adult human subjects. To ensure completeness, reference lists of relevant articles and previous reviews were also manually screened. Only publications in English were considered.

### The critical triangle: intestinal barrier, gut microbiota and immune system

**Fig. 1:**
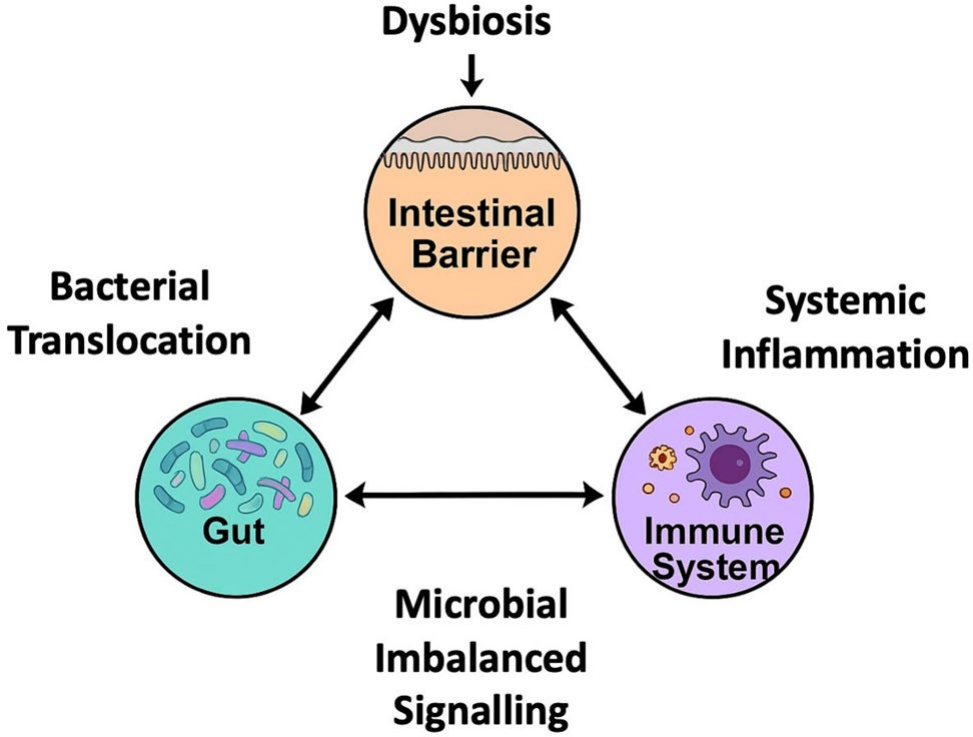
Critical triangle: intestinal barrier–gut microbiota–immune system in sepsis. Dysbiosis disrupts the intestinal barrier, increasing permeability and facilitating bacterial translocation (or bacterial products) into the systemic circulation. This promotes aberrant microbial signaling and fuels systemic inflammation, which, in turn, further compromises barrier integrity. The triangular interplay between gut microbiota, intestinal barrier, and immune system establishes a self-reinforcing cycle that amplifies sepsis pathophysiology

The gastrointestinal tract harbours the largest immune cell population of the human body. Its integrity depends on a finely tuned interaction between three interconnected elements: the intestinal barrier, the gut microbiota, and the host immune system. In health, these components form a protective triangle that maintains homeostasis by preventing microbial translocation, shaping immune responses, and ensuring tolerance to dietary components and commensal organisms [5]. In sepsis, however, this equilibrium is compromised [5–16]. Each side of the triangle is weakened, and their collapse amplifies systemic inflammation, fuelling organ dysfunction and shock (Fig. 1).

#### The intestinal barrier: the first line of defence

The intestinal barrier is not merely a passive wall, rather it is a complex, multi-layered defence system [24]. It includes luminal microbes, a mucus layer rich in antimicrobial peptides, an efficient gastrointestinal motility and secretion, a monolayer of epithelial cells connected by tight junctions, an underlying immune compartment populated by dendritic cells, lymphocytes, and macrophages, a gut vascular barrier and, finally, the liver barrier [24]. Together, these elements maintain selective permeability, allowing the absorption of nutrients while preventing the leakage of bacteria and toxins [25, 26].

During sepsis, this scenario is profoundly disrupted. Ischemia–reperfusion injury, cytokine storms, and oxidative stress damage epithelial cells and loosen tight junctions. Paneth cell dysfunction reduces antimicrobial peptide secretion, while mucus production diminishes [14]. The consequence is an increased intestinal permeability, often referred to as a “leaky gut”, which allows bacteria and microbial products, such as lipopolysaccharide (LPS), to enter the systemic circulation, perpetuating systemic inflammation [11].

#### The gut microbiota: from ally to reservoir of pathogens

Under physiological conditions, the gut microbiota plays a central role in maintaining barrier integrity. Commensals produce key metabolites, such as SCFAs, that strengthen epithelial tight junctions, regulate mucus secretion, provide energy to colonocytes and play a relevant role in maintaining the metabolic homeostasis [27]. Moreover, they compete with pathogenic species, preventing colonization and overgrowth [28].

Sepsis and critical illness rapidly dismantle this balance [21]. Environmental factors, antibiotic exposure, parenteral nutrition, and altered gut motility facilitate the onset of gut dysbiosis. Within days, microbial diversity collapses, beneficial anaerobes, such as *Bifidobacterium* and *Faecalibacterium prausnitzi*, decline, and opportunistic pathogens dominate [29, 30]. Beyond classical commensals, *Akkermansia muciniphila* has emerged as a next-generation probiotic with unique properties. This mucin-degrading bacterium actively promotes the renewal of the mucus layer, thereby reinforcing epithelial integrity and maintaining barrier function [31–33]. By stimulating mucus turnover, *A. muciniphila* not only preserves a critical defense against microbial translocation but also exerts anti-inflammatory and metabolic effects [31]. Preclinical studies have shown that its administration reduces systemic inflammation, improves glucose and lipid metabolism, and enhances gut barrier resilience under stress conditions [34]. These features make *A. muciniphila* a promising candidate for microbiome-targeted interventions in critical illness, potentially complementing or even surpassing the role of traditional probiotic strains [31]. Nevertheless, this protective potential is counterbalanced by the rapid rise of pathobionts under critical illness. *Enterococcus*, *Enterobacteriaceae*, and *Pseudomonas* often emerge as leading taxa, creating a reservoir for secondary infections [35–37].

This change shifts the gut from the site of symbiosis to that of predominant pathobionts which generate profound consequences [11]. Without SCFAs and other microbial metabolites, the epithelial barrier weakens further [20–22]. At the same time, overgrowth of pathobionts increases the load of PAMPs, which continuously stimulate innate immune receptors, such as toll-like receptors (TLRs). The resulting dysbiosis becomes a dominant contributing factor to systemic inflammation [11–15].

#### The immune system: crossroads of tolerance and hyperinflammation

The immune system is the third corner of this triangle, tightly intertwined with both the microbiota and the intestinal barrier [25, 38]. In homeostasis, gut microbiota primes the immune system to maintain tolerance, by promoting the development of regulatory T cells and suppressing excessive immune/inflammatory responses. Microbial metabolites modulate cytokine secretion, balancing pro- and anti-inflammatory signals [37]. Sepsis affects this delicate relationship [15]. Specifically, dysbiosis reduces the immune-derived regulatory signals, while the leaky barrier exposes immune cells to a flood of microbial antigens [14, 15]. The result is a paradoxical state of simultaneous hyperinflammation and immunosuppression. Excessive activation of TLRs and NOD-like receptors fuels cytokine storms, whereas immune exhaustion blunts the ability to control secondary infections. In this context, the immune system ceases to be a regulator and becomes over-reactive and ineffective, both features being hallmarks of sepsis [39].

### The triangle collapses: a vicious cycle

The breakdown of the intestinal barrier, the dysbiotic shift of the microbiota, and the immune dysfunction are not independent phenomena, rather they form a pathogenetically relevant vicious cycle [15]. Barrier disruption facilitates microbial translocation, which further stimulates immune activation. Immune dysregulation, in turn, worsens barrier injury through inflammatory mediators and ischemic damage [14, 15]. The dysbiotic microbiota amplifies both barrier dysfunction and immune imbalance [17]. This triangular collapse transforms the gut from a guardian into a driver of systemic pathology.

#### Beyond the triangle: systemic axes of communication

The implications of this collapse extend beyond the intestine. The gut–lung axis is increasingly recognized in the pathogenesis of sepsis-associated acute respiratory distress syndrome (ARDS) [40]. Translocation of microbial products primes alveolar macrophages, amplifying pulmonary inflammation. Similarly, the gut–brain axis may contribute to septic encephalopathy, with microbial metabolites and cytokines altering blood–brain barrier permeability and neural signalling [41]. These systemic extensions highlight how the failure of the intestinal triangle reverberates across distant organs, propagating the multi-organ dysfunction which is a prominent feature of sepsis.

#### Additional risks deriving from climate change and environmental pollution

Epidemiological and experimental data point to climate change [42, 43] and air pollution [44] as relevant risk factors for sepsis. In both cases, available evidence suggests a possible critical role played by gut microbiota and altered intestinal permeability.

Heat waves generated by climate-change increase the risk of hospitalization for several specific causes, including sepsis [42, 43]. Of note, significant variations in temperature, humidity and ultraviolet radiation can modulate the gut microbiota and to affect intestinal permeability [45–48]. Peripheral vasodilation secondary to heath waves can reduce blood flow in the gastrointestinal tract affecting the tight junctions and finally leading to increased intestinal permeability and endotoxemia [49–51].

Similar findings have been reported for air pollution. In a large US cohort, long-term PM_2.5_ exposure has been linked with increased risk of sepsis mortality [44]. In another Chinese cohort, short-term exposure to NO_2_ and O_3_ were positively associated with the risk of sepsis-related hospital admissions and stays [52]. In this scenario, epidemiologic and experimental studies documented significant associations between air pollution, variations in gut microbiota [53, 54], gut damage and altered gut permeability [55]. In particular, exposure to particulate matter can lower the expression of the tight junction proteins Claudin-1, Desmocollin and Zonula occludens-1 (ZO-1) [56], leading to increased gut permeability. In addition, the altered gut barrier function generated by PM exposure decreases the ability of gut macrophages to internalize and kill bacterial cells, also due to epithelial cell apoptosis and damage of tubulin cytoskeleton [57, 58]. An additional role derives from toxic chemicals contaminating ingested food and water, as pesticides. Pesticides can change the composition of gut microbiota [59] and affect gut permeability [60–62].

Similar effects are generated by microplastics [63–65]. In mice, exposure to polyethylene terephthalate microplastics affects the diversity and community composition of gut microbiota and the integrity of gut barrier [64]. Another animal model recently showed that exposure to polystyrene nanoplastics impairs the integrity of the gut barrier, compromising the expression of tight junction protein ZO-1 and mucin-13, and affecting the composition of gut microbiota [65]. Additional mechanisms can derive from a possible facilitating effect of microplastics on bacterial antimicrobial resistance [66].

### Innovative therapies and future options

Based on evidence indicating that unbalanced gut microbiota and altered gut barrier function play an active role in the trajectory of sepsis, therapeutic strategies aimed at restoring eubiosis may become a new frontier in critical care. The idea may sound speculative, but history reminds us that many of today’s standard therapies once began as provocative hypotheses. The collapse of the intestinal barrier–microbiota–immune system triangle offers multiple targets for intervention, ranging from restoration of microbial diversity to modulation of microbial metabolites (Table 1).

**Table 1:** Emerging therapeutic strategies targeting the gut microbiota in sepsis

Intervention	Mechanism of action	Evidence (preclinical/clinical)	Limitations/challenges
Faecal microbiota transplantation	Restores microbial diversity and barrier integrity	Promising animal data; case reports and pilot trials in humans	Safety concerns (donor screening, pathogen transfer), logistics, limited large RCTs
Probiotics	Replenish beneficial bacteria; modulate immune responses; enhance barrier function	Mixed results in clinical studies; some evidence in ICU prevention	Strain-specific effects; risk of bacteraemia in immunocompromised patients
Prebiotics	Promote growth of beneficial microbes; enhance SCFA production	Strong preclinical rationale; limited clinical evidence	Variable host response: optimal formulation and dosage unclear
Postbiotics	Deliver microbial-derived metabolites directly (e.g., SCFAs, bacteriocins)	Early experimental studies	Standardization, stability, and safety in critical illness
Synbiotics	Combined prebiotics and probiotics for synergistic effect	Some encouraging ICU trials, but data heterogeneous	Complex formulation; interaction with antibiotics
Targeted antibiotics/selective decontamination	Reduce pathogenic overgrowth; maintain commensals	Used in some ICUs; evidence of reduced infection rates	Risk of resistance; inconsistent global acceptance; ethical concerns
Microbial-derived metabolites	Direct administration of protective metabolites (e.g., butyrate)	Strong experimental rationale; very early stage	Delivery methods, pharmacokinetics, and systemic effects not well-characterized

*ICU* Intensive care unit, *RCT* Randomized controlled trial, *SCFA* Short-chain fatty acids

#### Faecal microbiota transplantation: a radical but promising approach

Faecal microbiota transplantation (FMT) represents the most direct attempt to restore microbial diversity. Originally developed for recurrent *Clostridioides difficile* infection (CDI), FMT has shown remarkable efficacy in re-establishing a healthy microbial ecosystem confirmed by several randomized controlled trials (RCTs) [67]. van Nood et al. showed that FMT following vancomycin pretreatment was superior to vancomycin alone for recurrent CDI, with cure rates over 80% for FMT vs. 31% for vancomycin alone [68]. More recent RCTs have confirmed and expanded these results, demonstrating that FMT is more effective than standard antibiotic therapy (vancomycin or fidaxomicin) for recurrent CDI, with efficacy rates ranging from 67% to over 90%, depending on delivery method and study design [69–71]. A 2023 Cochrane review identified six RCTs comparing FMT to antibiotics or placebo in immunocompetent adults with recurrent CDI, finding that FMT nearly doubled the likelihood of resolution compared to control [72]. Other RCTs have compared various FMT delivery routes (oral capsule, colonoscopy, enema) and found comparable efficacy, with colonoscopic and oral routes generally outperforming enema [73, 74]. For primary CDI, a 2025 multicentre RCT found that FMT was non-inferior to vancomycin as first-line therapy, with similar rates of clinical cure and recurrence prevention [75]. However, most guidelines and trials continue to focus on recurrent rather than initial CDI. These robust data provide the proof-of-concept that targeted modulation of the gut ecosystem can achieve clinically meaningful outcomes in humans, thereby legitimizing the exploration of FMT in other critical settings, including sepsis. Although clinical experience in sepsis remains limited, preclinical evidence is encouraging. In animal models, FMT reduced bacterial translocation, attenuated systemic inflammation, and improved survival [22, 76].

Clinical evidence in sepsis remains scarce but intriguing. Small case series have reported restoration of microbial diversity and improved gastrointestinal function after FMT in critically ill patients [77, 78]. These findings, while far from being definitive, highlight the need for structured trials to determine whether the benefits observed in preclinical models can be translated into improved outcomes in human sepsis.

While most available evidence on dysbiosis in sepsis derives from critically ill populations, an increasing number of studies highlight that patients hospitalized in Internal Medicine wards (where most septic patients are admitted) display profound baseline microbial alterations. Advanced age, multimorbidity, polypharmacy, chronic renal or hepatic dysfunction, and repeated antibiotic exposure contribute to reduced microbial diversity and overgrowth of pathobionts, such as *Enterococcus* or multidrug-resistant *Enterobacterales* [79–81]. These features have been associated with a higher risk of nosocomial and bloodstream infections, even in the absence of critical illness. Prospective observational studies have shown that dysbiosis in non-ICU patients predicts infection-related complications and mortality, suggesting that microbiome-oriented preventive strategies may be particularly beneficial in this population.

In addition, growing evidence indicates that the benefits of FMT extend beyond the treatment of recurrent CDI [72]. A prospective cohort study showed that patients receiving FMT for CDI recurrence experienced a markedly lower incidence of bloodstream infections during the following 90 days, with a 23% reduction in risk compared with those treated with antibiotics [82]. While this suggests a potential preventive impact on candidemia, the study did not specifically isolate candidemia as an endpoint, and direct evidence for candidemia prevention following FMT remains limited in the literature. These findings provide a proof-of-concept that restoring microbial homeostasis may enhance systemic antifungal and antibacterial resilience, offering promising opportunities for infection prevention in frail, high-risk hospitalized adults.

#### Probiotics, prebiotics, synbiotics, and postbiotics

A more targeted approach involves the use of probiotics, a term used to indicate live microorganisms intended to confer health benefits. Several randomized trials have tested probiotics in ICU patients, with mixed results [83, 84]. For instance, a recent meta-analysis in ICU patients demonstrated that probiotic administration was associated with a relative risk reduction of approximately 25–30% in ventilator-associated pneumonia, though results varied across strains and study designs. Similar, but less consistent, benefits were observed for bloodstream infections, with reductions ranging from 10 to 20% [85]. However, concern about safety, particularly the risk of bacteraemia or fungemia in immunocompromised patients, remains an open issue.

Prebiotics, i.e., non-digestible substrates that stimulate the growth of beneficial bacteria, offer another avenue [84–86]. By selectively nourishing commensals, such as *Bifidobacterium* or *Lactobacillus*, prebiotics may indirectly restore microbial balance [86, 87]. Synbiotics combine probiotics and prebiotics, aiming for synergistic effects [87, 88]. Evidence suggests that synbiotics can improve intestinal barrier function and reduce infection rates in surgical and ICU populations, though again, data in septic shock are limited [89].

An emerging concept is the use of postbiotics: microbial-derived metabolites or components that exert beneficial effects without requiring live organisms. SCFAs, such as butyrate, or tryptophan-derived indoles, have shown immunomodulatory and barrier-protective properties in experimental models [90, 91]. Postbiotics may bypass the risks associated with live organisms while delivering the key functional molecules of a healthy microbiota.

#### Personalized antibiotics and microbiome-friendly strategies

Antibiotics remain the cornerstone of sepsis management, yet they are also the most potent disruptors of the microbiome [1]. Broad-spectrum regimens, while lifesaving, accelerate dysbiosis and promote colonization by multidrug-resistant organisms [5]. This paradox creates an urgent need for more selective strategies. Personalized antibiotic therapy guided by microbial profiling is an emerging vision. Rapid sequencing technologies may allow clinicians to tailor antibiotics not only to pathogen susceptibility, but also to minimize collateral damage to beneficial commensals of gut microbiota. Narrow-spectrum agents, cycling protocols, or adjunctive protective strategies can help preserve microbial diversity [92].

Another potentially useful approach is “microbiome-friendly” critical care, such as the preferential use of enteral nutrition enriched with fermentable fibres, minimization of unnecessary systemic antibiotics, and integration of probiotic or prebiotic supplementation into ICU protocols, which may have profound long-term consequences for microbial resilience [93].

#### Emerging frontiers: phages, engineered microbes, and beyond

Beyond conventional strategies, several futuristic therapies are under investigation. Bacteriophage therapy, i.e., viruses that specifically target pathogenic bacteria, offers the possibility of eliminating harmful taxa while sparing commensals. Though still experimental, phages have shown promise in multidrug-resistant infections and may be integrated into future sepsis care [94–96].

Synthetic biology provides another exciting horizon. Engineered bacteria could be designed to deliver therapeutic metabolites, secrete anti-inflammatory molecules, or fight pathogens within the gut ecosystem. Similarly, nanoparticle-based delivery of microbial metabolites, such as butyrate, could provide targeted immunomodulation. These approaches remain speculative but illustrate the potential of microbiome-centred therapeutics [97–99].

#### Challenges and limitations

Despite the promising data, significant challenges remain. Safety is of paramount importance since introducing live organisms into critically ill, immunocompromised patients carries possible life-threatening risks. Standardization of therapies, such as FMT, is difficult, as donor variability and preparation methods profoundly influence outcomes. Regulatory pathways for microbiome-based therapies remain underdeveloped, and large-scale randomized trials in septic populations are still scarce. Another limitation is timing. Sepsis evolves rapidly, and the window for effective microbial intervention may be narrow. The feasibility of complex therapies, such as FMT, in the acute setting should be critically evaluated. Patient selection is equally challenging: the identification of microbiome signatures will help selecting patients who are most likely to benefit. Such tools are not yet ready for clinical use and represent one of the future areas of research.

### Future perspectives

The evolving understanding of the microbiota contribution to sepsis pathophysiology opens a new frontier for translational and clinical research. Yet, if this field is to meaningfully influence patient care, several conceptual and methodological shifts must occur. The future of sepsis management may be defined not only by hemodynamic monitoring, antimicrobial stewardship, and organ support, but also by the systematic integration of microbial ecology into bedside decision-making.

In addition, evidence pointing to a role for environmental factors as possible key-players in the onset of gut dysbiosis, altered gut barrier function and, in turn, in facilitating sepsis in subjects at risk should not be neglected. In this respect, besides future lines of research oriented toward therapeutic strategies and precision medicine, relevant results could derive from primary prevention strategies oriented to possibly mitigate climate changes and to lower the release of widely diffused toxics as air pollutants, pesticides, and microplastics.

#### Toward microbiome-stratified sepsis care

A central challenge is the extreme interindividual variability of the microbiome. Age, diet, genetics, comorbidities, prior antibiotic exposures, and geographic factors all shape the microbial landscape. This variability implies that “one-size-fits-all” interventions (whether probiotics, antibiotics or FMT) will likely fail. Future strategies should move toward microbiome-stratified approaches, in which patients are categorized according to defined microbial signatures or “dysbiosis phenotypes.” Such stratification could inform not only targeted therapies but also prognostication, allowing clinicians to anticipate which patients are at higher risk of rapid deterioration or refractory shock.

#### Harnessing multi-omics integration

Next-generation sequencing and advanced bioinformatics now allow simultaneous characterization of bacterial, viral, and fungal communities, as well as host transcriptomics, proteomics, and metabolomics. The future lies in integrating these data sets to identify actionable patterns: microbial taxa linked with specific immune signatures, metabolic profiles predictive of organ dysfunction, or virome shifts associated with immunosuppression [100]. In sepsis, a condition with high heterogeneity, multi-omics integration could finally deliver the precision medicine long promised but rarely realized [101, 102]. Artificial intelligence and machine learning will be indispensable tools to interpret these high-dimensional data and translate them into clinically usable algorithms [103].

#### Clinical trial design for microbiome oriented therapeutic strategies

A fundamental barrier is the lack of high-quality clinical trials directly testing microbiota-targeted interventions in sepsis [5]. Most studies remain preclinical or limited to small cohorts. Future trials should be based on designs that reflect the complexity of both microbiota and sepsis. Adaptive platform trials could be particularly valuable, allowing iterative testing of multiple microbiota-directed strategies within a single framework. Endpoints should extend beyond mortality to include trajectories of organ dysfunction, immune recovery, and microbiota resilience. Importantly, longitudinal sampling must become standard, enabling researchers to capture the dynamic evolution of microbiota–host interactions over the course of illness.

#### Therapeutic horizons: beyond the gut

While most attention has focused on intestinal ecology, future perspectives should embrace a broader view of the “holobiont”, the host with all its microbial partners. The lung microbiome, for instance, has been shown to undergo profound alterations during mechanical ventilation, potentially influencing ventilator-associated pneumonia and ARDS [40]. Similarly, the skin microbiome may impact barrier defence in critically ill patients. Therapies that integrate and coordinate interventions across multiple microbial niches (gut, lung, skin) may represent the next stage of innovation [104].

*Ethical and practical considerations.*

The integration of microbiota science into sepsis care will inevitably raise practical and ethical challenges. FMT, for example, remains logistically complex and carries unresolved concerns regarding donor screening and long-term safety. Moreover, interventions such as personalized antibiotic regimens guided by microbial profiling should compare cost, accessibility, and health equity. Without careful attention, advances in microbiota-driven sepsis care could exacerbate disparities between resource-rich and resource-limited healthcare settings.

#### A new paradigm for sepsis

Ultimately, the forefront of sepsis treatment may demand a reframing of sepsis itself. Rather than viewing it solely as an aberrant host response to infection, one may increasingly conceptualize it as a disorder of the environment–host–microbe ecosystem. This paradigm shift could have profound implications, encouraging clinicians and researchers to treat not just the patient, but also the living environment and the microbial communities that co-define their physiology.

The coming decade offers an extraordinary opportunity to move beyond description and correlation toward actionable, tailored and microbiome-informed interventions. If successful, the integration of microbiota science into sepsis care could provide not only better survival outcomes, but also recovery, resilience, and long-term health. The challenge now is to harness innovation without losing sight of feasibility, equity, and patient-centred outcomes. The microbiota is no longer a collection of germs with marginal effects, rather it emerges a critical partner influencing significantly humans’ health.

## Conclusion

Sepsis continues to represent a challenge in acute and critical care medicine. Despite decades of research, mortality remains high and therapeutic innovation has lagged. Increasingly, the gut microbiota appears as a missing piece in this puzzle, offering a new perspective on why some patients have an eventful consequence from septic shock, while others recover. Once regarded as a passive bystander, the intestinal microbiome is now understood as an active participant in immune regulation, barrier integrity, and systemic homeostasis. When disrupted, this complex ecosystem may betray its host, fuelling the vicious cycle of inflammation, immune paralysis, and organ failure.

The evidence remains preliminary, yet compelling enough to demand attention. Biomarkers of intestinal injury and permeability are gaining traction, while experimental therapies (ranging from faecal microbiota transplantation to precision-tailored probiotics) are beginning to challenge traditional paradigms. More importantly, the field is evolving from mere correlation toward causality, recognizing the microbiome as a potential therapeutic target rather than a passive indicator of disease severity. Looking ahead, the integration of microbiome science into sepsis management requires both ambition and caution. The ambition lies in embracing multi-omics approaches, machine learning, and innovative trial designs that can capture the heterogeneity of sepsis and microbial ecosystems. The caution lies in ensuring that interventions remain safe, equitable, and feasible in the complex reality of clinical care. In many ways, the microbiota represents a new frontier, a hidden organ whose health and resilience may determine the trajectory of sepsis. To ignore it would be to overlook a critical partner in survival. To embrace it could open the door to prevention strategies and to transformative therapies, reshaping how we diagnose, stratify, and ultimately manage one of the medicine’s most devastating syndromes.

The time has come to view sepsis not only as an infectious or immunological disorder, but as a disease of disrupted environment–host–microbe symbiosis. This concept will help to bridge the gap between pathophysiological insight and meaningful therapeutic progress, casting hope to patients who nowadays still face an uncertain fate.

## Abbreviations

ARDS: Acute respiratory distress syndrome
CDI: Clostridioides difficile infection
DAO: Diamine oxidase
FMT: Faecal microbiota transplantation
I-FABP: Intestinal fatty acid binding protein
ICU: Intensive care unit
PAMP: Pathogen-associated molecular patterns
RCT: Randomized controlled trial
SCFA: Short-chain fatty acids
TLR: Toll-like receptor
ZO-1: Zonula occludens-1
